# Diagnosing and Treating Intolerance to Carbohydrates in Children

**DOI:** 10.3390/nu8030157

**Published:** 2016-03-10

**Authors:** Roberto Berni Canani, Vincenza Pezzella, Antonio Amoroso, Tommaso Cozzolino, Carmen Di Scala, Annalisa Passariello

**Affiliations:** 1 Department of Translational Medical Science, University of Naples “Federico II”, 80131 Naples, Italy; cinzia.pezzella@gmail.com (V.P.); antonioamoroso87@gmail.com (A.A.); tom.cozzolino@gmail.com (T.C.); carmendiscala@gmail.com (C.D.S.); annalisa.passariello@unina.it (A.P.); 2 European Laboratory for the Investigation of Food Induced Diseases, University of Naples “Federico II”, 80131 Naples, Italy; 3 CEINGE Advanced Biotechnologies, University of Naples “Federico II”, 80131 Naples, Italy

**Keywords:** lactose intolerance, fructose malabsorption, sucrase-isomaltase deficiency, glucose-galactose malabsorption, sorbitol intolerance, trehalose intolerance, FODMAPs intolerance, breath test, molecular analysis

## Abstract

Intolerance to carbohydrates is relatively common in childhood, but still poorly recognized and managed. Over recent years it has come to the forefront because of progresses in our knowledge on the mechanisms and treatment of these conditions. Children with intolerance to carbohydrates often present with unexplained signs and symptoms. Here, we examine the most up-to-date research on these intolerances, discuss controversies relating to the diagnostic approach, including the role of molecular analysis, and provide new insights into modern management in the pediatric age, including the most recent evidence for correct dietary treatment.

## 1. Introduction

Adverse food reactions (AFR) represent a relevant problem in daily clinical practice, but are poorly recognized and managed. They are common in industrialized countries, where, depending on data collection methods and definitions, they affect up to 20% of the general population [1]. The prevalence increases significantly in patients with irritable bowel syndrome (IBS). It has been described that up to 80% of IBS patients believe that their symptoms are diet-related, of which three quarters are possibly related to intolerance to carbohydrates [2,3].

**Figure 1:**
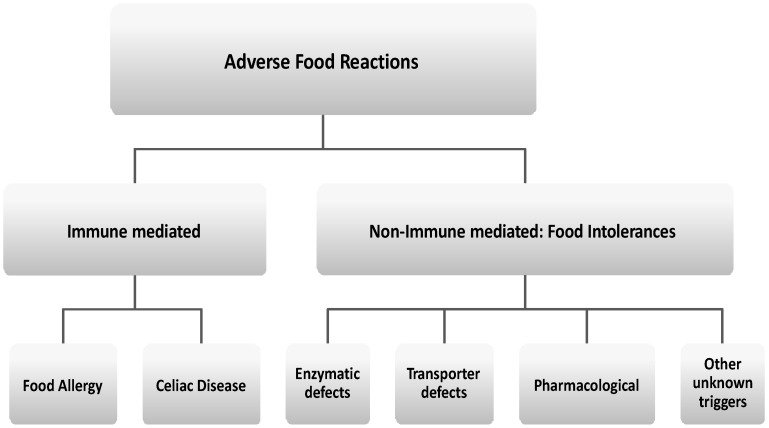
Classification of adverse food reactions (AFR).

According to the main pathophysiologic mechanism, AFRs are commonly classified into different groups (Figure 1) [4,5,6,7,8].

**Figure 2:**
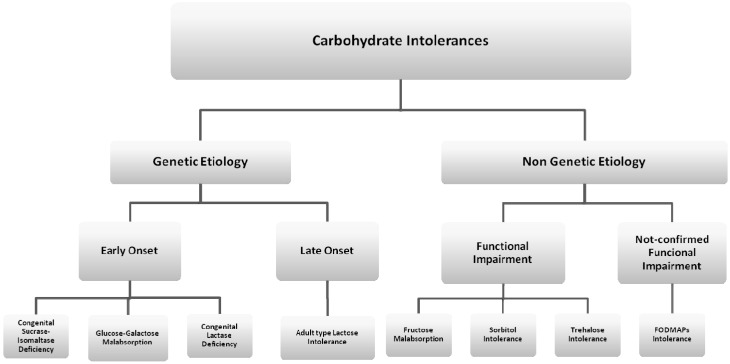
Classification of carbohydrate intolerances. CSID: Congenital Sucrase-Isomaltase Deficiency; GGM: Glucose-Galactose Malabsorption; CLD: Congenital Lactase Deficiency.

Intolerance to carbohydrates is the most common type of non-immune-mediated AFR (Figure 2). The prevalence of this condition has seemed to increase during the last few decades as a consequence of the growing rate of carbohydrate consumption in the diet. National survey data in the United States have indicated that, over the past few decades, consumption of selected carbohydrates, largely in the form of added sugars, has increased by up to 900% [9].

**Figure 3:**
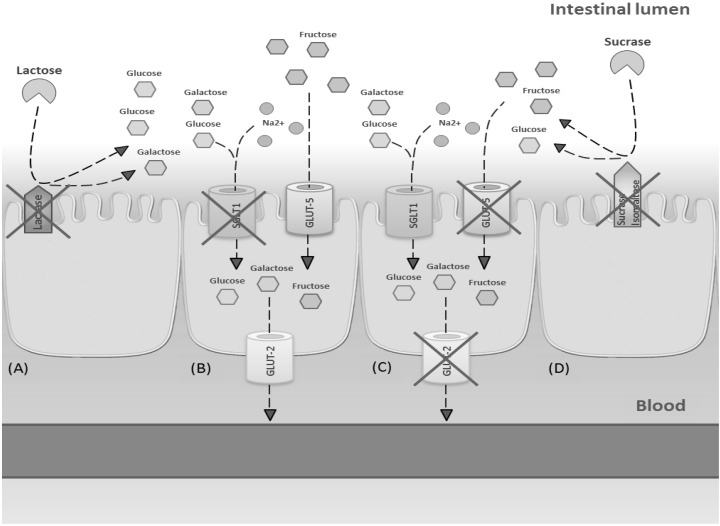
Mechanisms involved in main carbohydrate intolerances. (**A**) Lactose intolerance due to deficiency of lactase enzyme; (**B**) glucose-galactose malabsorption due to a genetic defect in SGLT1 expression; (**C**) fructose malabsorption due to dose-dependent transporters overloading; (**D**) sucrase malabsorption due to a genetic defect in sucrase-isomaltase activity.

Symptoms of intolerance to carbohydrates are primarily due to deficiency of enzymes or transporters or overloading of a transport system located on the brush border of the epithelium lining the small intestine (Figure 3). Non-absorbed carbohydrates in the intestinal tract drive fluids into the lumen through an osmotic force, causing osmotic diarrhea. Moreover, non-absorbed carbohydrates are fermented by gut microbiota to gas.

**Figure 4:**
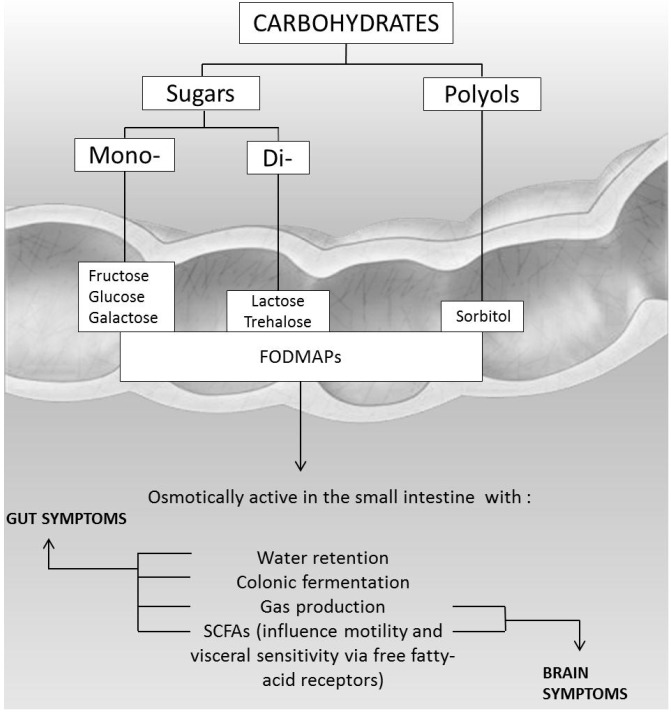
Pathogenesis of gut and brain symptoms in patients with intolerance to carbohydrates.

All these events are responsible for the clinical symptoms, such as distension of the small bowel, non-focal abdominal pain associated with bloating and flatulence, nausea, increased gut motility, and diarrhea [2,10,11,12,13,14]. Extraintestinal symptoms, such as headache, vertigo, memory impairment, and lethargy have been described in less than 20% of subjects with carbohydrate intolerance [15,16]. These systemic symptoms could be the result of toxic metabolites, produced by sugar fermentation of colonic bacteria, that can alter cell signalling mechanisms (Figure 4).

These manifestations frequently lead to investigations to rule out other organic disorders at intestinal and extra-intestinal levels, including invasive procedures. Thus, there is a large unmet need for a clear diagnosis as well as consistent and effective advice on dietary treatments for these conditions. There have been significant advances in understanding the scientific basis of carbohydrate intolerances, and new forms (e.g., Fermentable Oligosaccharides, Disaccharides, Monosaccharides, and Polyols intolerance, and non-celiac gluten sensitivity) have been identified [1,14]. Intolerance to carbohydrates often begins in childhood. Here, we examine the most up-to-date research on childhood intolerance to carbohydrates, discuss controversies relating to the diagnostic approach, including the role of molecular analysis, and provide new insights into the modern management of these conditions.

## 2. Genetic Etiology with Early Onset Carbohydrate Intolerances

### 2.1. Congenital Sucrase-Isomaltase Deficiency (CSID)

Congenital sucrose-isomaltase deficiency (CSID, OMIM #222900) is a rare autosomal recessive inherited disease of the small intestine resulting from genetic mutations in sucrase-isomaltase, an enzyme complex responsible for catalyzing the hydrolysis of dietary sucrose and starch [17]. Decreased or absent sucrase and/or isomaltase enzymatic activity has been found in patients with CSID, and investigations at the subcellular and molecular levels in intestinal biopsy specimens have led to the description of several phenotypes, differing in transport efficiency, processing, and sorting of the protein, which result in impaired physiologic functions [17,18,19,20]. In addition to the degree of enzyme deficiency, the appearance of overt clinical manifestations of CSID is partially determined by the amount of sugar and starch consumed [21]. The prevalence in the European population has been estimated at 1 in 5000, but it is higher among the indigenous populations of Alaska, Greenland, and Canada [22]. Gastrointestinal symptoms usually begin after an infant is weaned off breast milk and is first exposed to sucrose and starch [23]. Failure to absorb dietary disaccharides and starch has implications for the absorption of other nutrients and the hormonal regulation of gastrointestinal function. For these reasons, patients with CSID are at risk for chronic malnutrition and failure to thrive. In many cases, the symptoms of CSID are more severe in infants than in adults. It has been suggested that an increased susceptibility to symptoms in infants is related to a shorter length of the small intestine. Although intestinal biopsy is still adopted in many tertiary centers for CSID diagnosis, genetic testing is now widely available. Molecular genetics has become helpful for obtaining an early and unequivocal diagnosis in infants with chronic diarrhea due to any of a variety of different disorders, thus permitting rapid and targeted therapeutic strategies and reducing repetitive, invasive, and expensive procedures. At least 80% of CSID patients have one of four common mutations [22,24]. Three of these four mutations (p.Val577Gly, p.Gly1073Asp, and p.Phe1745Cys) are in the sucrase domain and have been identified previously for their role in cellular trafficking. They have been confirmed to abolish sucrase and isomaltase activity. The fourth mutation, p.Arg1124X, in the isomaltase domain, introduces a chain termination codon and interrupts the amino acid coding sequence [24]. Other diagnostics are also available, but less used in clinical practice, such as sucrose breath hydrogen tests [25] and intestinal disaccharidases activity measurement on biopsy specimens [20]. In the treatment of CSID, the administration of an oral solution containing sacrosidase (Sucraid) as enzyme replacement therapy could be helpful, along with dietary restriction (Table 1). This enzyme, derived from the *Saccharomyces cerevisiae*, is generally well tolerated in patients with CSID and induces a reduction of symptoms by helping sucrose digestion [21,26].

### 2.2. Glucose-Galactose Malabsorption

Glucose-galactose malabsorption (GGM, OMIM 606824) is a rare autosomal recessive disorder caused by a defect in the solute carrier family 5 member 1 gene SLC5A1, which codes for a Na+/glucose co-transporter [27]. This transporter is responsible for the tight coupling of two Na+ ions and one glucose or galactose molecule across the membrane of the epithelial cells lining the small intestine and renal proximal tubule [28]. The prevalence of this condition is still unknown, because only a few hundred cases have been described. Patients with congenital GGM present with severe, life-threatening chronic diarrhea. The malabsorbed glucose and galactose, and derived short chain fatty acids (SCFAs), reaching the colon determine osmotic diarrhea. Many patients present an improvement of symptoms in adulthood, because of a better absorption of sugars, but the underlying mechanism is unclear. A tentative diagnosis of GGM is based on the following criteria: (1) onset of diarrhea soon after birth; (2) evidence of carbohydrate malabsorption with positive reducing substance in the stool; (3) failure to improve with lactose free and amino acid based formula; (4) strong improvement of diarrhea only with elimination of glucose and galactose; (5) exclusion of infections. The diagnosis can be confirmed by molecular analysis of the SLC5A1 gene [29,30,31,32]. More than 40 mutations of SLC5A1 responsible for GGM have been described, but a real genotype/phenotype correlation is still lacking. This modern approach has replaced other diagnostic tests such as hydrogen breath test with glucose or galactose [33], or the oral glucose/galactose tolerance test [34]. Patients with GGM improve their symptoms with a specific low concentration of glucose-galactose in the diet and using a fructose formula in early life (Table 2).

## 3. Genetic Etiology with Late-Onset Carbohydrate Intolerances

### Lactose Intolerance

According to the origin, lactose intolerance can be classified into three main forms:

1. Congenital lactase deficiency: a rare autosomal recessive disease where enzymatic activity is absent or reduced from birth;
2. Secondary lactase deficiency: a transient condition deriving from intestinal damage secondary to small bowel bacterial overgrowth, infections, celiac disease, Crohn’s disease, or radiation enteritis;
3. Adult type lactase deficiency: an autosomal recessive condition resulting from a developmentally regulated change of the lactase gene product, responsible for reduced synthesis of the precursor protein.

Congenital lactase deficiency (CLD, MIM 223000) is a very rare (only a few cases have been described) and severe form of lactase deficiency in which this enzymatic activity is very low or absent from birth [35]. The main symptoms are watery diarrhea, meteorism, and malnutrition, beginning on the first days after birth with the onset of lactation. Symptoms disappear when patients change to a lactose-free diet. The typical feature of CLD is very low levels of lactase-phlorizin hydrolase (LPH), the enzyme responsible for the digestion of lactose. The activities of other disaccharidases and the histological structure of the epithelium of the small intestine are normal [36]. Most CLD cases have been described in Finland, where the disorder is enriched due to a founder effect and genetic drift [36]. This is in contrast to adult-type hypolactasia (where lactase activity declines after weaning), which is common all over the world [36]. Premature stop codons and a truncated protein as a result of frame shifts, missense mutations in the coding region of LPH, or exon duplication are the most common genotypes identified in CLD patients [35,36,37,38]. Some other cases include mutations leading to single amino acid substitutions that can interfere with the proper maturation and function of LPH [36,39]. Recently, severe forms of CLD elicited by mutations in the LPH gene that occur in either a compound heterozygous or homozygous pattern of inheritance have been described [40].

**Figure 5:**
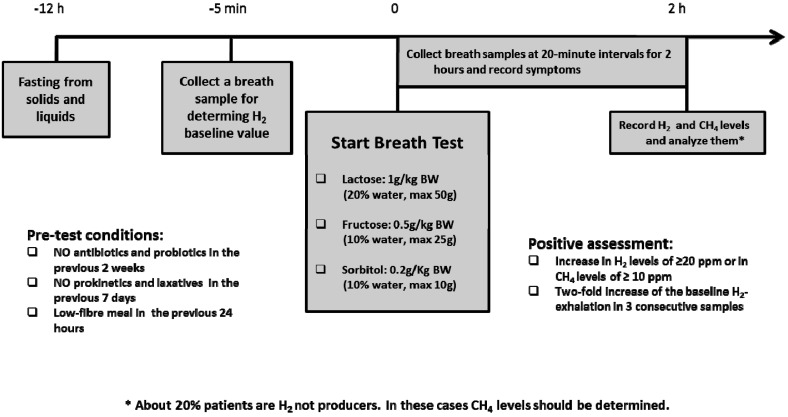
Breath test procedures in children with suspected carbohydrate intolerances.

In about 70% of the worldwide general population, LPH activity decreases below a critical threshold between the ages of two and five years; this is the most frequent cause of enzyme deficiency [12,41,42]. The rate of adult type lactase deficiency varies among ethnic groups (e.g., Asia 80% to 100%, Africa 70% to 95%, USA 15% to 80%, Europe as a whole 15% to 70%) and is based on the non-persistence of LPH after childhood [42]. The persistence or non-persistence (hypolactasia) of the expression of LPH is associated with the point polymorphism C/T 13910. This consists of a substitution in a sequence of DNA that regulates the LPH gene: genotype CC correlate with hypolactasia, while TT genotype with lactase persistence [43]. Several individual factors influence the development of symptoms in non-persistence lactase subjects: dose of lactose in diet, oro-cecal transit time, lactase expression, distribution and fermentation capacity of gut flora [44], sensitivity towards chemical and mechanical stimulation of the gut, and psychological factors [45,46]. Adaptation of intestinal microbiota, assuming a growing dose of lactose, with increase of bacterial b-galactosidase activity is recognized as a cause of reduction of the symptoms of lactose intolerance [47,48]. Anamnesis and hydrogen breath test are the mainstay of adult-type lactose intolerance diagnosis (Figure 5).

Another available diagnostic test is the lactose tolerance test (LTT), in which a patient suspected of lactose intolerance assumes 50 g of lactose dissolved in water. Samples of capillary blood are taken to test the plasma glucose concentration at −5, 0, 15, 30, 45, and 60 min. A maximal plasma-glucose increase of 1.4 mmol/L or higher indicates lactose tolerance [49]. A meta-analysis comparing the diagnostic accuracy of lactose breath hydrogen or lactose tolerance tests found that the overall sensitivity was 0.88 (confidence interval [CI], 0.85–0.90) and the specificity was 0.85 (CI, 0.82–0.87) for the breath test. The lactose tolerance test showed a sensitivity of 0.94 (CI, 0.9–0.97) and a specificity of 0.90 (CI, 0.84–0.95) [50]. The genetic test, identifying single nucleotide polymorphism associated with lactase persistence/non-persistence, is also available. It should be noted that the presence of the lactase non-persistent gene does not imply the simultaneous presence of lactose intolerance that may appear later in the life. More recently, a test based on the measurement of D-xylose after lactase cleavage of orally administered 4-galactosylxylose (Gaxilose) has been investigated in a large multicenter study in adults, with good sensitivity and specificity for lactase deficiency as determined in biopsy specimens [51].

Management of lactose intolerance consists in the avoidance of all lactose-containing foods (Table 3 and Table 4) [52]. In adult-type hypolactasia, dairy products are generally avoided for 2–4 weeks, the time required for remission of symptoms. Then, a gradual reintroduction of dairy products low in lactose up to a threshold dose of individual tolerance should be recommended. In secondary hypolactasia, a restricted diet is necessary only for a limited time period. [53]. Available data suggest that adults and adolescents with a diagnosis of lactose intolerance could ingest up to 12 g of lactose in a single dose (equivalent to the lactose content in 1 cup of milk) without any symptoms or with only minor symptoms [53]. Products containing lactic acid, lactalbumin, lactate, and casein do not contain lactose, so they can be consumed [53]. The oral administration of Beta-Galactosidase represents another possible therapeutic approach for the treatment of primary lactase deficiency. The data showed an improvement of gastrointestinal symptoms and a decrease of H_2_ levels at breath test with the administration of 1500 U/day of Beta-Galactosidase. However, data regarding the efficacy of this microbial exogenous enzyme are still needed [54] Yogurt with live cultures is generally well tolerated by individuals with lactose intolerance.

Subjects with lactose intolerance could be at risk of lower calcium intake, so calcium supplementation is required and the recommendation of calcium fortified foods should be considered. The current recommendations for calcium intake are 700 mg/day for children aged 4 to 9 years, and 1300 mg/day over 10 years, according to EFSA guidelines [55].

## 4. Non-Genetic Etiology Carbohydrate Intolerances

### 4.1. Fructose Malabsorption

Fructose is a six–carbon monosaccharide molecule naturally present in a great variety of daily foods, such as fruits, vegetables, and honey [56]. It is also produced through enzymatic processing of corn as high fructose corn syrup (HFCS), which is increasingly used in the food industry as a cheaper, tasteless, readily available sweetener in many products, such as sodas, candies, and artificial fruit juices [57]. Furthermore, this monosaccharide is also present as disaccharide, the sucrose, in complex with glucose [58]. The main fructose carriers, GLUT-5 and GLUT-2, two members of the glucose transport family (GLUT), provide for the passive uptake of fructose: GLUT-5, located on the brush border membrane of human small intestine enterocytes, is a glucose-independent transporter with low, saturable uptake capacity; GLUT-2 is a high-capacity, glucose-dependent fructose co-transporter. In addition to fructose, GLUT-2 is also a transporter for glucose and galactose, constitutively located on the basolateral membrane. In specific conditions, GLUT-2 can be expressed on the apical membrane. Fructose malabsorption should not be confused with hereditary fructose intolerance (a metabolic disease whose incidence is estimated to be 1 in 25,000 individuals) in which a lack of functional aldolase B results in an accumulation of fructose-1-phosphate in the liver, kidneys, and intestine [57], causing hypoglycemia, nausea, bloating, abdominal pain, diarrhea, and vomiting [57]. A useful test for diagnosis of fructose malabsorption is the hydrogen breath test [58], by which the H_2_ produced is measured noninvasively in collected samples of expired breath after the ingestion of a standardized dose of 0.5 g/kg of fructose to a maximum of 25 g dissolved in water (sensitivity and specificity both 80% to 90%) [14]. The diagnosis is confirmed by an increase of ≥20 ppm in H_2_ or ≥10 ppm in CH_4_ levels over the baseline twice in succession and abdominal discomfort after the consumption of the test dose. However, according to other authors, a negative breath test result does not exclude a positive response to fructose restriction, so the hydrogen breath test does not seem to be the appropriate diagnostic means to predict the response to the diet [59]. Fructose malabsorption can be secondary to intestinal injury (induced by several diseases such as celiac disease) [60,61,62,63]. The rate of children who tested positive for malabsorption on a fructose breath test is significantly higher in younger age groups (<9 years) [64]. It was hypothesized that the rapid decrease of the fructose malabsorption from infancy to higher age could reflect the normal developmental maturation of the mechanisms of fructose absorption [64]. Moreover, the importance of fructose malabsorption is hypothesized to depend on the ratio of fructose to glucose, but the specific mechanism responsible has yet to be clearly elucidated [62]. In fact, the active reabsorption of glucose from the small intestine is due to the transport system SGLT-1/sodium-glucose cotransporter. When the ingested glucose is transported by SGLT1, GLUT2 is activated and inserted into the apical membrane, so the co-ingestion of glucose greatly enhances fructose absorption [58,65,66,67,68,69,70,71,72,73,74]. A second mechanism has been postulated: fructose with other solutes is absorbed by a paracellular transport system, based on the opening of tight junctions induced by glucose absorption [58]. These mechanisms with glucose could explain the reason why, when the concentration of fructose in a certain food is present in excess of glucose concentration, some individuals may develop fructose malabsorption [72,73,74]. On the other hand, sorbitol seems to have a negative effect on fructose absorption. This sugar alcohol (polyol) can be transformed into fructose, blocking GLUT-5 and leading to aggravation of the fructose uptake disorder [75]. The treatment of fructose malabsorption is based on a reduction of fructose intake lower than 10 g/day, and the elimination of sugar alcohols and alcoholic beverages (Table 5). Moreover, it is essential to educate patients about the importance of a balanced intake of fructose and glucose, for the reasons described above. Furthermore, the intake of xylose isomerase as a dietary supplement, increasing the conversion of fructose to glucose, seems to ameliorate the symptoms of fructose malabsorption [76]. Using these dietetic strategies, it is possible to obtain remission of symptoms in 60% to 90% of cases [76].

### 4.2. Sorbitol Intolerance

Sorbitol is a carbohydrate naturally present in fruits and juices. It is also used in commercially products such as drugs, sweets, dietetic foods, and chewing gum. Sorbitol absorption is dose and concentration related. Sorbitol H_2_ breath test is effective in detecting small bowel damage with a relevant reduction of absorption surface, but it is not specific for any condition responsible for intestinal malabsorption [14]. A diet with low sorbitol concentration is indicated in Table 6.

### 4.3. Trehalose Intolerance

Trehalose is a disaccharide composed of two glucose molecules and found in mushrooms and algae [77]. Intestinal trehalose, a brush border enzyme, is a beta-galactosidase that catalyzes the hydrolysis of trehalose to two glucose molecules for absorption. It is present throughout the small intestine, with the highest levels in the proximal jejunum [77]. Isolated trehalose deficiency represents an autosomal dominant condition, and occurs in at least 8% of Greenland’s population [58]. Nevertheless, only three cases have been reported elsewhere, two of whom were first-degree relatives. Up to now, only a study by Arola is available about trehalose malabsorption and H_2_ breath testing [58]. In this work, a 25 g oral trehalose load test was performed in 64 subjects. Trehalase activity was determined in serum and on a duodenal biopsy specimen and symptoms of intolerance were recorded. Intolerant subjects were best differentiated from tolerant subjects by changes in breath gases (hydrogen and methane) and duodenal trehalase/sucrase ratio. The change in breath gases correlated inversely with duodenal trehalase activity [58]. Nevertheless, no conclusive evidence is available to support trehalose H_2_ breath testing in clinical practice and, therefore, the performance of this test is not recommended [63].

### 4.4. FODMAPs Intolerance

Fermentable oligosaccharides, disaccharides, monosaccharides, and polyols (FODMAPs) are a group of short-chain carbohydrates poorly absorbed at the intestinal level. A list of high FODMAPs foods is reported in Table 7.

These highly osmotic substances are fermented by gut bacteria and may induce gastrointestinal symptoms by luminal distention or through direct action at the intestinal level by an as-yet undefined mechanism [78]. The therapeutic approach to FODMAPs intolerance is based on an exclusion diet (Table 8). Considering the high number of foods included in the FODMAPs list and that some people may be more sensitive to some groups of FODMAPs than others, the exclusion diet should be carefully tailored by an expert pediatric nutritionist based on the clinical history and the results of selected sugars breath tests [79]. The FODMAPs free diet is usually recommended for 4–6 weeks. Following this period of elimination, patients are encouraged to “challenge” themselves with different groups of FODMAPs, in order to determine which group of FODMAPs they are sensitive to, and then to liberalize the diet as much as possible.

The challenge phase can be done either by adding foods high in a particular group of FODMAPs for a day, or by starting with a very small amount of FODMAPs from one group and gradually adding more items into the diet in order to determine the individual tolerance. If there is little efficacy after six weeks of elimination, the diet should be discontinued. However, some patients who report inadequate symptom improvement with the diet still report that their symptoms are aggravated when they eat high-FODMAPs foods [79].

## 5. Conclusions

Intolerance to carbohydrates is common in childhood. The pathophysiologic mechanisms are variable, leading to different onset age and management. The therapeutic approach is based on dietary treatment that should be supervised by an experienced nutritionist who can tailor the diet according to the necessities of the patient limiting the risk of malnutrition. Genetics is providing new insights through molecular analysis for early diagnosis of severe forms of intolerance to carbohydrates.

## Figures and Tables

**Table 1:** Low sucrose diet.

**Foods to avoid**
Cereals with added sugars Apple, apricot, banana, cantaloupe, grapefruit, melon, mango, orange, peach, pineapple, tangerine Carrot, potato Beans, chickpeas, green peas, lentils, peas, soy Yogurt sweetened with sucrose, sweetened condensed milk, sweetened cream Sugar (sucrose), ice cream, all desserts made with sugar, marmalade, candies, jellies, chocolate, licorice, commercial cookies and cakes with added sugar, sweetened drinks
**Foods allowed**
Wheat, rice, corn, einkorn, oats, kamut, spelt, rye, bread, pasta, flour, cereals with no added sugar * Avocado, berries, cherries, fig, grapes, kiwi, lemon, lime, olives, papaya, pear, pomegranate, prune, strawberries All vegetables Milk, dairy product, butter, cream, cheeses, yogurt sweetened with dextrose or fructose All meat, fish, and eggs All fats Fructose, honey, cocoa, unsweetened juice, homemade low-sucrose cookies and cakes

* In patients who can ingest starch at 40–50 grams per serving two to three times per day.

**Table 2:** Low glucose and galactose diet.

**Foods to avoid**
All kinds of milk, butter, yogurt, cheeses and other dairy products Sugar (sucrose), ice cream, all desserts made with sugar, candies, gelatin desserts, chocolate, licorice, commercial cookies and cakes with added sugar, sweetened drinks Glucose, dextrose, dextrin, maltose, maltodextrin, corn syrup, glucose polymers, lactose, stevia
**Foods allowed**
Special formula without galactose and glucose Small amounts of: pasta, rice, potatoes, bread, unsweetened cereal, puffed wheat, puffed rice, oat, wholegrain cereals without a sugary coating, quinoa All legumes (beans, chickpeas, peas, lentils, soy) All vegetables All fruits All meat, fish, and eggs All vegetable fats Fructose, honey, cocoa, sugar-free marmalade, unsweetened juice, all fructose-sweetened desserts and snacks

**Table 3:** Lactose content in common dairy foods.

Food	Lactose (g/100 g of Food)
Skimmed cow’s milk	4.7
Low-fat cow’s milk	4.6
Whole cow’s milk	4.5
Buttermilk	4.1
Free lactose milk	0.5
Whole powdered milk	35.1
Skimmed powdered milk	50.5
Goat’s milk	4.2
Buffalo milk	4.9
Yogurt	3.2
Butter	4.0
Cottage cheese	2.6
Mozzarella cheese	1.5–2.0
Goat cheese	1.5–2.0
Ricotta cheese	4.0
Parmigiano Reggiano	0–0.9
Cream cheese	6.0
Taleggio cheese	0
Fontina cheese	0
Provolone cheese	0
Gorgonzola cheese	0

**Table 4:** Low lactose diet.

**Foods to limit**
All kinds of milk: whole, low fat, nonfat, cream, powdered, condensed, evaporated, goat, acidophilus, and chocolate Butter, cottage cheese, ice cream, creamy/cheesy sauces, cream cheeses, soft cheeses (brie, ricotta), mozzarella, whipped cream, yogurt Fish and meat (breaded or creamed) Milk bread, crackers, creamed, scalloped, or au gratin potatoes Muffin, biscuit, waffle, pancake, and cake mixes; milk chocolate; bakery products and desserts that contain the ingredients listed above
**Foods allowed**
Lactose-free milk, soy milk Lactose-free dairy, hard cheeses (Parmigiano Reggiano, Pecorino, Grana Padano, fontina, taleggio, provolone, Swiss), gorgonzola All fruits All vegetables All legumes All cereals All meat, fish, and eggs All vegetable fats

**Table 5:** Low fructose diet.

**Foods to Avoid**
All fruits Broccoli, carrots, cauliflower, green beans, green peppers, sweet potatoes, tomatoes Beans, peas Corn Fructose, honey, high-fructose corn syrup, sorbitol, jams, gelatin desserts, candies, all desserts sweetened with fructose Condiments such as barbeque sauce, ketchup, sweet and sour sauce, pancake syrup, plum sauce, chutney, *etc.*
**Foods Allowed**
Asparagus, celery, chives, cucumber, kale, lettuce, parsnips, pumpkin, radish, scallions, spinach, spinach, white potatoes, shallots, zucchini All cereals All meat, fish, and eggs All dairy All fats Sugar (sucrose), molasses, saccharine

**Table 6:** Low sorbitol diet.

**Foods to Avoid**
Apple, apricot, blackberry, cherry, date, fig, nectarine, pear, peach, plum, raisin, and other dried fruits Sugar-free chewing gum and candies Diabetic foods and drinks Diet and light drinks Foods that contain the initials E420 in the list of ingredients
**Foods Allowed**
Banana, citrus fruit, kiwi, melon, pineapple, strawberry All legumes All cereals All vegetables All meat, fish, and eggs Milk and dairy products All fats Sugar, honey, fructose, cocoa, jams, gelatin desserts, molasses, all desserts made with sugar, marmalade, chocolate, commercial cookies and cakes with added sugar, sweetened drinks, artificial sweeteners: aspartame, saccharine, mannitol, isomalt, xylitol (cough drops, gums, mints)

**Table 7:** Main high FODMAPs foods.

Fructose	Lactose	Fructans	Galactans	Polyols
Fruits: watermelon, apple, mango, pear Jam Fruit juice Dried fruits Honey and molasses	Cow, sheep and goat milk Yogurt Cheese Mascarpone	Vegetables: asparagus, chicory, beets, broccoli, Brussels sprouts, cabbage, eggplant, fennel, onions, garlic, leeks Cereals: wheat, bread, biscuits, crackers, couscous, pasta Fruits: persimmon, watermelon	Legumes: beans, chickpeas, lentils	Fruits: apple, apricot, cherrys, lychee, peach, pear, plum, watermelon Vegetables: cauliflower, capsicum, mushrooms, corn Sweeteners: sorbitol, mannitol, maltitol, xylitol

**Table 8:** Low FODMAPs diet.

**Foods to limit**
Milk (from cow, sheep, or goat), butter, cottage cheese, ice cream, creamy/cheesy sauces, sweetened condensed milk, soft cheeses (brie, ricotta), mozzarella, whipped cream, yogurt Legumes (beans, chickpeas, peas, lentils) Wheat, einkorn, emmer, kamut, spelt, rye Artichokes, asparagus, beets, leeks, broccoli, Brussels sprouts, cabbage, cauliflower, fennel, green beans, mushrooms, garlic, onions Avocado, apples, apricots, dates, canned fruit, cherries, dried fruits, figs, mango, pears, papaya, peaches, plums, prunes, persimmon, watermelon Honey, jams, jellies, molasses, artificial sweeteners: sorbitol, mannitol, isomalt, xylitol (cough drops, gum, mints)
**Foods allowed**
Lactose-free milk and lactose-free dairy, hard cheeses (cheddar, Parmesan, pecorino, Swiss) Wheat-free grains: rice, corn, quinoa, tapioca, buckwheat Banana, berry, cantaloupe, grape, grapefruit, kiwi, lemon, lime, mandarin, orange, pineapple Bell peppers, cucumbers, carrots, celery, eggplant, lettuce, leafy greens, olives, pumpkin, potatoes, tomatoes, zucchini, most spices and herbs Sugar All fats
